# Causal relationship between the blood immune cells and intervertebral disc degeneration: univariable, bidirectional and multivariable Mendelian randomization

**DOI:** 10.3389/fimmu.2023.1321295

**Published:** 2024-01-10

**Authors:** Chaofan Qin, Mingxin Chen, Qingshuai Yu, Xin Wang, Tao Hu, Bo Lei, Zhengjian Yan, Si Cheng

**Affiliations:** Department of Orthopedics, Second Affiliated Hospital, Chongqing Medical University, Chongqing, China

**Keywords:** immune cells, intervertebral disc degeneration, multivariable mendelian randomization, univariable mendelian randomization, bidirectional mendelian randomization

## Abstract

**Background:**

Intervertebral disc degeneration (IVDD) is a prominent contributor to chronic low back pain, impacting millions of individuals annually. Current research on disc degeneration is placing a growing emphasis on the role of the immune system in this process. Nevertheless, the precise relationship between immunity and disc degeneration remains to be fully elucidated.

**Method:**

We obtained GWAS data for immune cells from the latest summary-level GWAS, including 6,620 individuals from Sardinian and 746,667 individuals from five global populations. Summary results for IVDD were sourced from the FinnGen consortium, comprising 20,001 cases and 164,682 controls. We conducted a comprehensive univariable Mendelian randomization (MR) analysis to explore the potential causal relationship between immune cells and IVDD. Primary estimation was carried out using Inverse-Variance Weighting (IVW). To ensure robustness, we employed additional MR methods such as MR-Egger, Weighted Median, Weighted Mode, and Simple Mode. Various tests were employed to assess pleiotropy and heterogeneity, including the Cochran Q test, leave-one-out test, MR-Egger intercept analysis and MR-PRESSO test. To account for potential confounding factors among the immune cells, we conducted a multivariable MR analysis. Finally, we investigated the possibility of a reverse association between immune cells and IVDD through bidirectional MR.

**Result:**

In total, our study identified 15 immune cells significantly associated with IVDD through univariable MR. Among these, 9 immune cell types were indicated as potential contributors to IVDD, while 6 were found to have protective effects. Importantly, we observed no evidence of heterogeneity or pleiotropy, signifying the robustness of our results. To mitigate confounding among immune cells, we utilized multivariable MR, leading to the discovery that only 9 immune cell types exerted independent effects on IVDD. These encompassed 7 as risk factors and 2 as protective factors. Additionally, our analysis revealed a bidirectional causal relationship between CD39+ CD4+ T cell %CD4+ T cell and IVDD.

**Conclusion:**

Our findings suggest a connection between immune cells and the risk of IVDD, shedding light on potential therapeutic avenues for modulating immune cell function in individuals with IVDD. However, the specific underlying mechanisms warrant further investigation in future experiments.

## 1 Introduction

Intervertebral disc degeneration (IVDD) is a prevalent degenerative disease, with an incidence of 40-50 cases per 10,000 people (1). IVDD is a pathological condition that can lead to persistent back pain or neurological deficits. The incidence of IVDD is gradually increasing, posing an escalating healthcare burden and economic pressure (2, 3). Unfortunately the etiology of IVDD still remains unclear (4–6). It is reported that intervertebral disc belongs to the immune-privileged tissue (7–9). Extrinsic factors, such as external forces and mechanical loads, play a role in annulus fibrosus rupture, with the subsequent immune response triggered by nucleus pulposus antigen exposure serving as the initiator of disc degeneration (10–13). Nevertheless, the association between immune cells and IVDD remains unconfirmed due to the absence of randomized controlled trials (RCTs).

Mendelian Randomization (MR), a method examining potential causal relationships between exposure factors and disease outcomes, has gained popularity in recent years (14). Consequently, we chose to employ univariable, multivariable, and bidirectional MR approaches to identify potential protective and risk factors for IVDD. This study focused on exploring the possible causal links between immune cells and IVDD, with the aim of uncovering novel targets for IVDD prevention and treatment.

## 2 Materials and methods

### 2.1 Study design

**Figure 1:**
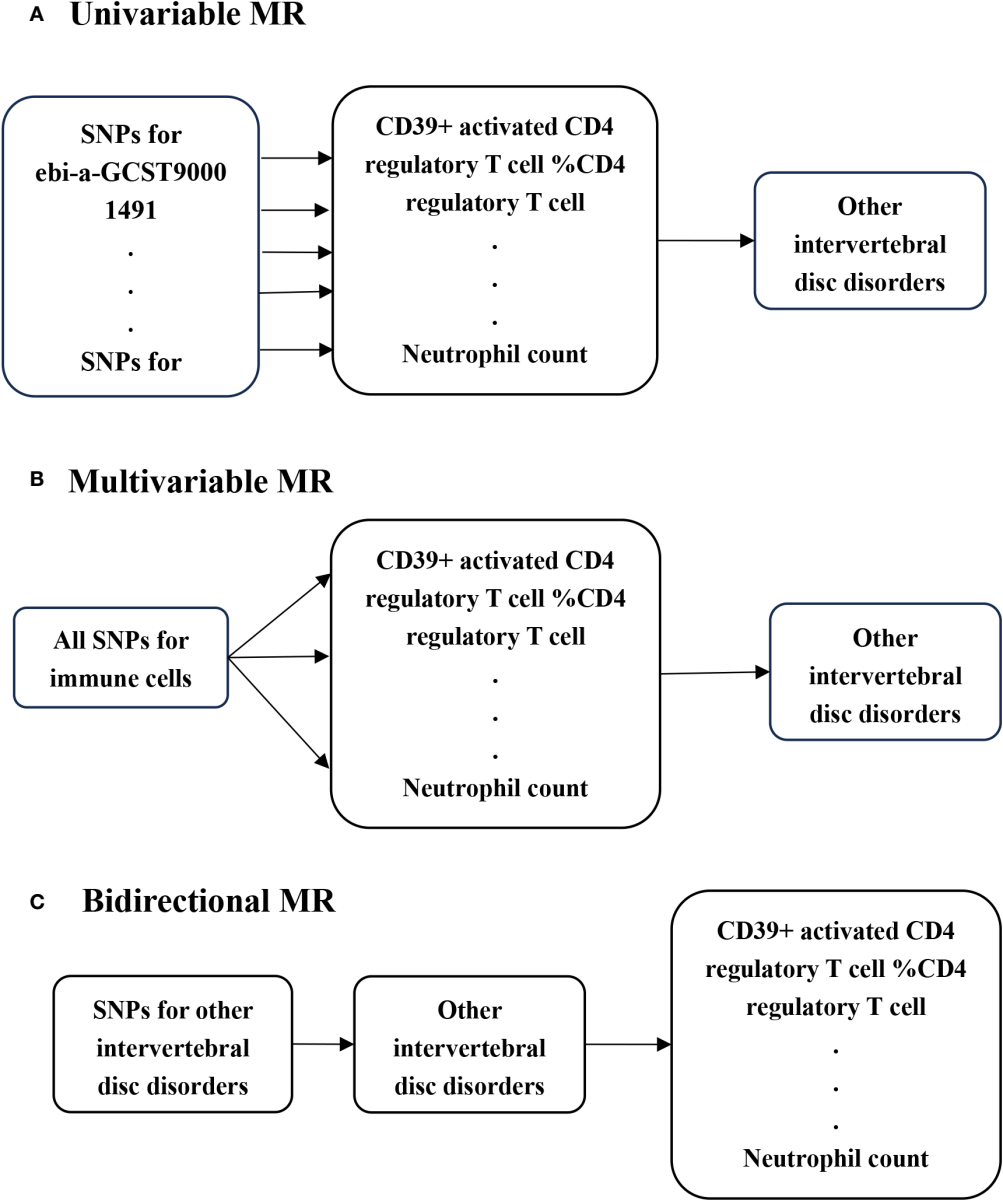
Schematic presentation of **(A)** univariable; **(B)** multivariable; **(C)** bidirectional.

In this study, we conducted univariable MR to investigate potential causal associations between various immune cells and IVDD, utilizing genome-wide association studies (GWAS) summary statistics (**Figure 1A**). To ensure unbiased causal effects, MR analysis must adhere to three core assumptions: (I) the relevance assumption, where genetic variants are strongly associated with the exposure of interest; (II) the independence assumption, ensuring genetic variants are not associated with potential confounders; and (III) the exclusion restriction, stipulating that genetic variants affect outcomes solely through the exposure of interest (15). To further explore the link between immune cells and IVDD, we employed multivariable MR, assessing the independent beta of different immune cells, as these cells can influence each other’s effector values (**Figure 1B**). Additionally, we conducted bidirectional MR to investigate the possibility of reverse causality between the exposure and the outcome (14) (**Figure 1C**).

### 2.2 GWAS data source

The GWAS data on immune cells were obtained from the latest summary-level GWAS, which involved 6,620 individuals from Sardinian descent within the European population and 746,667 individuals from five diverse global populations (16, 17). The former study comprised 3,757 cases and 3,027 controls, with a gender distribution of 43% males and 57% females, and age ranges spanning from 18 to 102 years (16). The latter dataset included 408,961 individuals of European ancestry from the UK Biobank (UKBB), 143,988 individuals of Japanese ancestry from the Biobank of Japan (BBJ), and 5,275 African Americans from the Vanderbilt University Biobank (BioVU) (17).

The summary results for Intervertebral Disc Degeneration (IVDD) were acquired from the FinnGen consortium, specifically from the R9 release (https://www.finngen.fi/fi). This dataset encompasses 20,001 cases and 164,682 controls. IVDD diagnoses were based on the International Classification of Diseases, specifically ICD-10 (M51), ICD-9 722, and ICD-8 725 coding standards.

### 2.3 Instrumental variable selection

Genetic instrumental variables for immune cell exposures were carefully chosen based on a genome-wide significance threshold (p<5×10^–8^) (14). We employed the PLINK clumping method to assess the linkage disequilibrium (LD) among the selected SNPs, with LD defined as SNPs having an R^2^>0.001 and being within a 10,000 kb physical distance (14). SNPs identified to be in LD were subsequently excluded from further analysis. Additionally, to eliminate weak instrumental variables, we calculated F-statistics for the SNPs using a previously described approximation method (18, 19). Importantly, all included exposures exhibited F-statistics exceeding 10 [**Supplementary Table 1**].

### 2.4 Statistical analysis

We primarily employed the inverse-variance weighted (IVW) method as our main approach in Mendelian randomization (MR). In addition, we conducted MR-Egger, weighted median, weighted mode, and simple mode analyses to ensure the robustness of our results. To assess heterogeneity among summary estimates, Cochran’s Q test was utilized. Moreover, we utilized the MR-Egger intercept to address and account for pleiotropy (14). To further assess the robustness of our findings, we implemented a leave-one-out analysis. The MR-PRESSO test was employed to identify and address pleiotropy, and to remove outliers (20).

We conducted all statistical analyses using the “TwoSampleMR” (version 0.5.7) packages and MR-PRESSO package within the R statistical software (version 4.3.1). A significance level of p < 0.05 was chosen to denote statistical significance.

## 3 Result

### 3.1 Causal effect from blood immune cells on intervertebral disc degeneration

We undertook a comprehensive Mendelian randomization study aimed at uncovering the causal relationship between 744 immune cell types and IVDD [**Supplementary Table 2**]. In our analysis, we identified 24 specific blood types of immune cells that demonstrated a causal association with disc degeneration through univariable MR [**Supplementary Table 3**], including Memory B cell %B cell, Naive-mature B cell %B cell, IgD-CD38dim B cell %lymphocyte, CD39+activated CD4 regulatory T cell %CD4 regulatory T cell, CD39+ secreting CD4 regulatory T cell %CD4 regulatory T cell, CD45RA+ CD8+ T cell %CD8+ T cell, CD8+ T cell %leukocyte, CD39+CD4+ T cell %T cell, CD39+ CD4+ T cell %CD4+ T cell,CD39+ CD8+ T cell %T cell, CD39+ CD8+ T cell Absolute Count, CD127- CD8+ T cell Absolute Count, CD28- CD8+ T cell Absolute Count, CD19 on IgD+ CD38+ B cell, CD19 on IgD+ CD38dim B cell, CD19 on IgD- CD38- B cell, CD19 on unswitched memory B cell, CD19 on transitional B cell, CD24 on IgD+ CD38+ B cell,CD28 on CD39+ secreting CD4 regulatory T cell, CD25 on CD39+ CD4+ T cell, CD4 on CD39+ CD4+ T cell, CD4 on CD39+ resting CD4 regulatory T cell and Neutrophil count. We excluded certain exposures due to a limited number of available SNPs to prevent potential bias, including Memory B cell %B cell, Naive-mature B cell %B cell, IgD-CD38dim B cell %lymphocyte, CD8+T cell %leukocyte, CD127-CD8+ T cell Absolute Count, CD19 on IgD-CD38-B cell, CD24 on IgD+ CD38+ B cell, CD28 on CD39+ secreting CD4 regulatory T cell and CD4 on CD39+ resting CD4 regulatory T cell.

**Figure 2:**
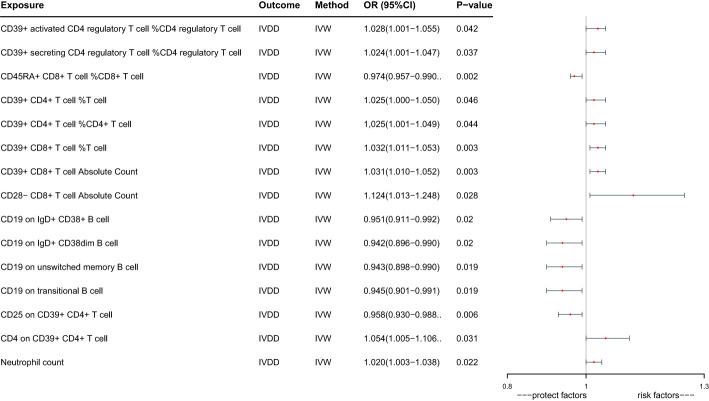
Forest plots of effects of immune cells on the risk of IVDD by using univariable MR.

Ultimately, we identified 15 immune cells that have a potential causal relationship with IVDD (**Figure 2**). CD39+ activated CD4 regulatory T cell %CD4 regulatory T cell increase the risk of IVDD significantly (OR: 1.028, 95%CI:1.001-1.055, P=0.042), CD39+ secreting CD4 regulatory T cell %CD4 regulatory T cell increase the risk of IVDD significantly (OR: 1.024, 95%CI:1.001-1.047, P=0.037), CD45RA+ CD8+ T cell %CD8+ T cell decrease the risk of IVDD significantly(OR:0.974,95%CI: 0.957 -0.990, P=0.002),CD39+ CD4+ T cell %T cell increase the risk of IVDD significantly (OR: 1.025, 95%CI:1.000-1.050, P=0.046), CD39+ CD4+ T cell %CD4+ T cell increase the risk of IVDD significantly (OR:1.025, 95%CI:1.001-1.049, P=0.044), CD39+ CD8+ T cell %T cell increase the risk of IVDD significantly (OR:1.032, 95%CI:1.011-1.053,P=0.003), CD39+ CD8+ T cell Absolute Count increase the risk of IVDD significantly (OR:1.031, 95%CI:1.010-1.052,P=0.003), CD28- CD8+ T cell Absolute Count increase the risk of IVDD significantly (OR:1.124, 95%CI:1.013-1.248, P=0.028), CD19 on IgD+ CD38+ B cell decrease the risk of IVDD significantly (OR:0.951,95%CI:0.911-0.992,P=0.020), CD19 on IgD+ CD38dim B cell decrease the risk of IVDD significantly (OR:0.942, 95%CI:0.896- 0.990,P=0.020), CD19 on unswitched memory B cell decrease the risk of IVDD significantly(OR:0.943,95%CI:0.898 -0.990, P=0.019), CD19 on transitional B cell decrease the risk of IVDD significant (OR:0.945, 95%CI:0.901-0.991,P=0.019), CD25 on CD39+ CD4+ T decrease the risk of IVDD significantly (OR:0.958,95%CI:0.930 -0.988,P=0.006), CD4 on CD39+ CD4+ T cell increase the risk of IVDD significantly (OR:1.054, 95%CI:1.005-1.106,P=0.031), Neutrophil count increase the risk of IVDD significantly (OR:1.020, 95%CI:1.003-1.038, P=0.022).

**Figure 3:**
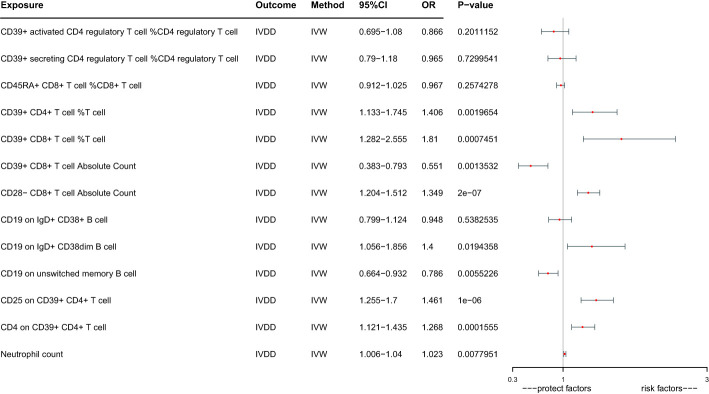
Forest plots of effects of immune cells on the risk of IVDD by using multivariable MR.

Given the interrelated nature of various immune cells, they have the potential to act as confounding factors for each other. Hence, we employed multivariable MR to estimate the independent direct effect of each exposure on the outcome. Our analysis revealed that only 9 immune cell types maintained an independent role when accounting solely for the effect of each exposure on the outcome (**Table 1**; **Figure 3**). CD39+ CD4+ T cell %T cell increase the risk of IVDD significantly (OR:1.406, 95%CI: 1.133-1.745). CD39+ CD8+ T cell %T cell increase the risk of IVDD significantly (OR: 1.810, 95%CI: 1.282-2.555). CD39+ CD8+ T cell Absolute Count decrease the risk of IVDD significantly (OR: 0.551, 95%CI: 0.383-0.793). CD28- CD8+ T cell Absolute Count increase the risk of IVDD significantly (OR: 1.394, 95%CI: 1.204-1.512). CD19 on IgD+ CD38dim B cell increase the risk of IVDD significantly (OR: 1.400, 95%CI: 1.056-1.856). CD19 on unswitched memory B cell decrease the risk of IVDD significantly (OR: 0.786, 95%CI: 0.664-0.932). CD25 on CD39+ CD4+ T cell increase the risk of IVDD significantly (OR: 1.461, 95%CI: 1.255-1.700).CD4 on CD39+ CD4+ T cell increase the risk of IVDD significantly (OR:1.268, 95%CI: 1.121-1.435). Neutrophil count increases the risk of IVDD significantly (OR: 1.023, 95%CI: 1.006-1.040). After controlling for confounding factors, the remaining six immune cell types no longer exhibit independent effects. Interestingly, in the aftermath of multivariate Mendelian Randomization, CD39+ CD8+ T cell Absolute Count, CD19 on IgD+ CD38dim B cell, and CD25 on CD39+ CD4+ T cell exhibited opposing effects on IVDD (**Figures 2**, **3**).

**Table 1:** Multivariable MR immune cells on IVDD.

exposure	outcome	b	pval	or	or_lci95	or_uci95
CD39+ activated CD4 regulatory T cell %CD4 regulatory T cell	IVDD	-0.144	0.2011152	0.866	0.695	1.080
CD39+ secreting CD4 regulatory T cell %CD4 regulatory T cell	IVDD	-0.035	0.7299541	0.965	0.790	1.180
CD45RA+ CD8+ T cell %CD8+ T cell	IVDD	-0.034	0.2574278	0.967	0.912	1.025
CD39+ CD4+ T cell %T cell	IVDD	0.341	0.0019654	1.406	1.133	1.745
CD39+ CD8+ T cell %T cell	IVDD	0.593	0.0007451	1.810	1.282	2.555
CD39+ CD8+ T cell Absolute Count	IVDD	-0.596	0.0013532	0.551	0.383	0.793
CD28- CD8+ T cell Absolute Count	IVDD	0.300	0.0000002	1.349	1.204	1.512
CD19 on IgD+ CD38+ B cell	IVDD	-0.054	0.5382535	0.948	0.799	1.124
CD19 on IgD+ CD38dim B cell	IVDD	0.336	0.0194358	1.400	1.056	1.856
CD19 on unswitched memory B cell	IVDD	-0.240	0.0055226	0.786	0.664	0.932
CD25 on CD39+ CD4+ T cell	IVDD	0.379	0.0000010	1.461	1.255	1.700
CD4 on CD39+ CD4+ T cell	IVDD	0.238	0.0001555	1.268	1.121	1.435
Neutrophil count	IVDD	0.023	0.0077951	1.023	1.006	1.040

**Figure 4:**
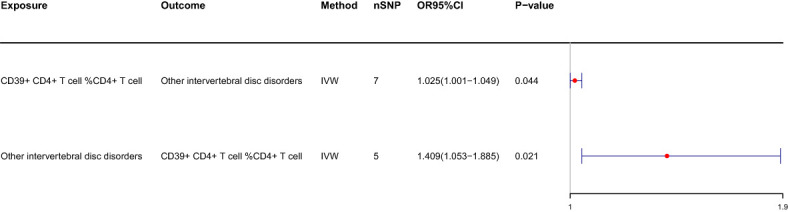
Forest plots of the bidirectional MR between CD39+ CD4+ T cell % CD4+ T cell and IVDD.

We conducted a reverse MR analysis and identified reverse causality specifically between CD39+ CD4+ T cells %CD4+ T cells and IVDD. Additionally, we observed that other intervertebral disc disorders significantly increase the risk of CD39+ CD4+ T cell %CD4+ T cell (OR: 1.409, 95%CI: 1.053-1.885, p=0.021) (**Table 2**; **Figure 4**). The causal relationship between these factors appears to be bidirectional.

**Table 2:** bidirectional MR: IVDD and CD39+ CD4+ T cell %CD4+ T cell.

exposure	outcome	beta	pval	or	or_lci95	or_uci95
IVDD	CD39+ CD4+ T cell %CD4+ T cell	0.343	0.021	1.409	1.053	1.885
CD39+ CD4+ T cell %CD4+ T cell	IVDD	0.024	0.044	1.025	1.001	1.049

### 3.2 Sensitivity analyses

**Figure 5:**
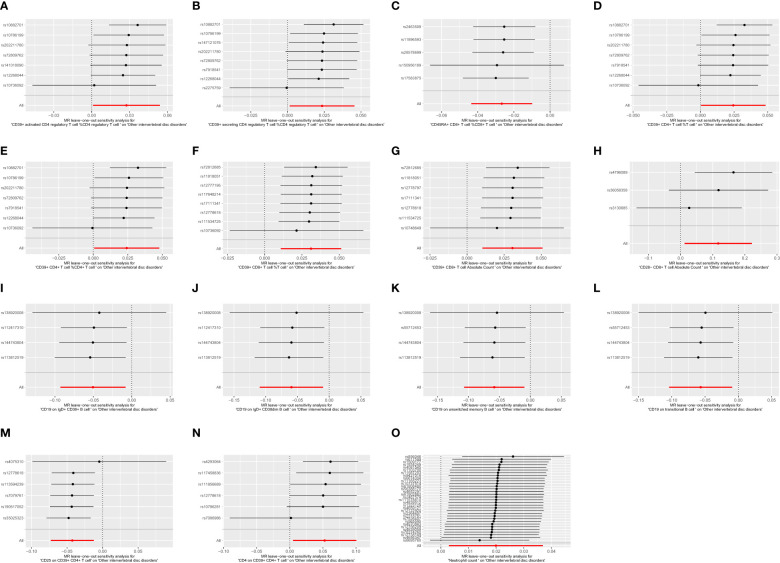
The leave one out plot of Mendelian randomization analyses. **(A)**:CD39+ activated CD4 regulatory T cell %CD4 regulatory T cell; **(B)**:CD39+ secreting CD4 regulatory T cell %CD4 regulatory T cell; **(C)**:CD45RA+ CD8+ T cell %CD8+ T cell; **(D)**:CD39+ CD4+ T cell %T cell; **(E)**:CD39+ CD4+ T cell %CD4+ T cell; **(F)**:CD39+ CD8+ T cell %T cell; **(G)**:CD39+ CD8+ T cell Absolute Count; **(H)**:CD28- CD8+ T cell Absolute Count; **(I)**:CD19 on IgD+ CD38+ B cell; **(J)**:CD19 on IgD+ CD38dim B cell; **(K)**:CD19 on unswitched memory B cell; **(L)**:CD19 on transitional B cell; **(M)**:CD25 on CD39+ CD4+ T cell; **(N)**:CD4 on CD39+ CD4+ T cell; **(O)**:Neutrophil count.

To ensure the robustness of our MR estimates, we conducted several sensitivity analyses. We employed Cochran’s Q test, MR-Egger intercept test, and a leave-one-out test. Notably, all MR-Egger intercept tests yielded P-values greater than 0.05, indicating the absence of pleiotropy. Additionally, the Cochran’s Q test revealed no significant heterogeneity between immune cells and IVDD (**Table 3**). Furthermore, the results remained stable in the leave-one-out test (**Figure 5**). The MR-PRESSO analysis revealed the absence of outliers and pleiotropy, as illustrated in **Table 3**. However, the MR-PRESSO test could not be applied to the CD28-CD8+ T cell Absolute Count due to the limited number of SNPs. In summary, the results of the univariable MR analysis are deemed acceptable.

**Table 3:** Results of sensitivity analysis of blood immune cells on the risk of intervertebral disc disorder.

exposure	MR-Egger intercept test		Cochran’s Q test		MR-PRESSO	
	egger_intercept	pval	Q	Q_pval	outlier	pval
CD39+ activated CD4 regulatory T cell %CD4 regulatory T cell	-0.0085	0.5446	9.1977	0.1628	0	0.347
CD39+ secreting CD4 regulatory T cell %CD4 regulatory T cell	-0.008	0.5198	9.272	0.2337	0	0.33
CD45RA+ CD8+ T cell % CD8+ T cell	-0.0127	0.3106	3.15	0.533	0	0.322
CD39+ CD4+ T cell %T cell	-0.0069	0.6116	9.0618	0.1701	0	0.87
CD39+ CD4+ T cell %CD4+ T cell	-0.0073	0.5885	8.9881	0.1743	0	0.498
CD39+ CD8+ T cell %T cell	-0.0045	0.7841	3.5504	0.8299	0	0.778
CD39+ CD8+ T cell Absolute Count	-0.0038	0.8173	3.6318	0.7264	0	0.34
CD28- CD8+ T cell Absolute Count	0.2286	0.4289	2.1451	0.3421	NA	NA
CD19 on IgD+ CD38+ B cell	0.0118	0.8656	0.2756	0.9645	0	0.353
CD19 on IgD+ CD38dim B cell	0.0097	0.8849	0.2482	0.9695	0	0.976
CD19 on unswitched memory B cell	0.0055	0.9307	0.2458	0.9699	0	0.971
CD19 on transitional B cell	0.0083	0.9015	0.2929	0.9614	0	0.599
CD25 on CD39+ CD4+ T cell	0.0518	0.2063	3.6186	0.6055	0	0.979
CD4 on CD39+ CD4+ T cell	-0.0012	0.9647	30.1944	0.4557	0	0.984
Neutrophil count	-0.0001	0.9827	30.1949	0.5072	0	0.751

NA, Not Available.

**Figure 6:**
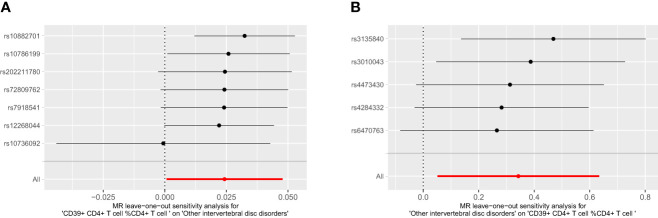
The leave one out plot of bidirectional MR between CD39+ CD4+ T cell % CD4+ T cell and IVDD. **(A)**: CD39+ CD4+ T cell %CD4+ T cell, **(B)**: other intervertebral disc disorder.

For the bidirectional MR analysis between CD39+ CD4+ T cell %CD4+ T cell and other intervertebral disc disorders, both the p-values of Cochran’s Q and MR-Egger intercept were greater than 0.05 (**Table 4**), suggesting the absence of heterogeneity and pleiotropy. The leave-one-out test reaffirmed the stability of these results (**Figure 6**). Pleiotropy and outliers were not observed in our results (**Table 4**). In summary, the bidirectional results remained stable.

**Table 4:** Results of sensitivity analysis between other intervertebral disc disorder and the risk of CD39+ CD4+ T cell %CD4+ T cell.

exposure	MR-Egger intercept test		Cochran’s Q test		MR-PRESSO	
	egger_intercept	pval	Q	Q_pval	outlier	pval
CD39+ CD4+ T cell %CD4+ T cell	-0.0073	0.5885	8.9881	0.1743	0	0.498
other intervertebral disc disorder	0.0565	0.558	3.6913	0.4494	0	0.501

## 4 Discussion

In our comprehensive study, we conducted MR to investigate the causal relationship between 744 immune cells and IVDD. Our analysis identified 15 immune cells significantly associated with IVDD, encompassing 6 protective factors and 9 risk factors. We utilized multivariable MR to eliminate the potential confounding factors, revealing that only 9 immune cells, including 7 risk factors and 2 protective factors. Furthermore, a bidirectional causal relationship between IVDD and CD39+ CD4+ T cell %CD4+ T cell was established through bidirectional MR analysis.Our results indicated that immune cells also played protective factors for IVDD. These findings provided a brand new understanding of the relationship between immunity and IVDD.

First, we observed a significant relationship between CD39+ CD4+ T cell %T cell, CD39+ CD8+ T cell %T cell, CD4 on CD39+ CD4+ T cell, CD25 on CD39+ CD4+ T cell, and CD39+ CD8+ T cell Absolute Count with IVDD. CD39 plays a crucial role in the immune system. CD39+ CD4+ T cells are linked to the effector functions of T helper (Th)17, known for their role in fostering chronic inflammation and contributing to matrix degradation (21–23). The collaboration of CD39 and CD73 leads to the conversion of ATP to ADP and cAMP, ultimately generating adenosine (24). Adenosine can interact with multiple receptors, such as A1, A2A, A2B, and A3, inducing various immune responses (25). Adenosine stimulates immune responses through A1 and A3 receptors, while it has immunosuppressive effects when interacting with A2A and A2B receptors (25–27). These findings might be the reasons that CD39+ CD4+ T cell %T cell, CD39+ CD8+ T cell %T cell, CD4 on CD39+ CD4+ T cell and CD25 on CD39+ CD4+ T cell can accelerate the degeneration and CD39+ CD8+ T cell Absolute Count plays a protective role in IVDD. However, further exploration of underlying mechanisms is needed.

Furthermore, our findings indicated associations between CD19 on unswitched memory B cells and CD19 on IgD+ CD38dim B cells with IVDD. CD19 is essential for B-cell maturation and function (28, 29). Reduced CD19 expression can result in decreased immune function (30). CD19 is increasing in the patient of IVDD (31). However, unswitched memory B cells lack the ability to produce antibodies, making them incapable of initiating an immune attack on the disc. CD38 facilitates inflammation, cell migration, phagocytosis, and antigen presentation (32). CD19 on IgD+ CD38dim B cells appears to activate the immune response to the disc, thereby contributing to IVDD.

In our study, we found that a type of immune cell count called CD28- CD8+ T cells could increase the risk of IVDD.CD28 usually helps activate CD8 cells to boost the immune response (33). Surprisingly, even without CD28, CD8+ still contributes to IVDD (34). So we suppose that CD8 plays a more crucial role and CD28 might not be the main factor in IVDD.

Adjusted by multivariate MR, CD39+ CD8+ T cell Absolute Count and CD25 on CD39+ CD4+ T cells changed from protective to facilitating factors. CD19 on IgD+ CD38dim B cells changed from facilitating to protective factors. This may be due to the fact that the interference between immune cells leads to the masking of their true role for IVDDD. Consequently, it was only through multivariate MR that we unveiled their true independent effects.

We also discovered a bidirectional potential causal link between CD39+ CD4+ T cell %CD4+ T cell and IVDD. It is suggested that there is a vicious cycle between immune cells and IVDD. This is a consequence of the immune system being initiated by the deteriorated disc and the activated immune system have irreversible damage to the disc (10, 12, 35).

Our MR analysis offers several advantages. Firstly, we employed univariable, multivariable, and bidirectional MR, mitigating confounding factors and reverse causality. Secondly, we conducted numerous sensitivity analyses to validate our hypotheses and minimize bias. Thirdly, we addressed population stratification issues by restricting the test population.

However, our study has limitations. It may not be directly applicable to other populations. The sample size was limited, potentially introducing bias, necessitating larger samples for robust results. Through we have utilized the univariable MR, bidirectional MR and multivariable MR, there is also unmeasured pleiotropy. Lastly, we refrained from using multiple analysis corrections as our primary goal was to identify potential biomarkers or therapeutic targets for IVDD. We opted not to apply the Bonferroni correction in our analysis since our primary focus was to identify potential therapeutic and prophylactic targets associated with IVDD. Bonferroni’s criteria are quite stringent and could have resulted in the exclusion of meaningful indicators.

## 5 Conclusion

Our comprehensive MR analysis revealed 15 immune cells associated with IVDD risk through univariable MR. After accounting for confounding effects between immune cells, our multivariable MR identified only 9 immune cells as independent factors for IVDD, comprising 7 risk factors and 2 protective factors. These 9 immune cells can serve as potential biomarkers for IVDD, offering new perspectives on its treatment and prevention. Nonetheless, further experiments are needed to elucidate the underlying mechanisms.

## Data availability statement

The datasets presented in this study can be found in online repositories. The names of the repository/repositories and accession number(s) can be found in the article/**Supplementary Material**.

## Ethics statement

Ethical approval was not required for the study involving humans in accordance with the local legislation and institutional requirements. Written informed consent to participate in this study was not required from the participants or the participants’ legal guardians/next of kin in accordance with the national legislation and the institutional requirements.

## Author contributions

CQ: Formal analysis, Software, Data curation, Investigation, Resources, Validation, Visualization, Writing – original draft. MC: Writing – review & editing, Investigation, Software, Validation. QY: Methodology, Project administration, Writing – review & editing. XW: Software, Writing – review & editing, Supervision. TH: Writing – review & editing, Investigation, Software. BL: Investigation, Writing – review & editing, Resources. ZY: Writing – review & editing, Conceptualization, Methodology, Project administration. SC: Writing – review & editing, Conceptualization, Formal analysis, Methodology, Software.
